# Premature Coronary Artery Disease, Association with Relative Blood Number of Mitochondrial DNA Copies in Mexican Population—Results of GEA Study

**DOI:** 10.3390/biology15181572

**Published:** 2026-09-08

**Authors:** Rosalinda Posadas-Sánchez, Marco Sánchez-Guerra, Citlalli Osorio-Yáñez, Giovanny Fuentevilla-Alvarez, Guillermo Cardoso-Saldaña, José Manuel Fragoso, Gilberto Vargas-Alarcón

**Affiliations:** 1 Departamento de Endocrinología, Instituto Nacional de Cardiología Ignacio Chávez, Mexico City 14080, Mexico; rosalinda.posadas@cardiologia.org.mx (R.P.-S.); miguel.fuentevilla@cardiologia.org.mx (G.F.-A.); guillermo.cardoso@cardiologia.org.mx (G.C.-S.); 2 Instituto Nacional de Perinatología, Mexico City 14080, Mexico; masanguerra@gmail.com; 3 Laboratorio de Fisiología Cardiovascular y Trasplante Renal, Unidad de Investigación en Medicina Traslacional, Instituto de Investigaciones Biomédicas, Universidad Nacional Autónoma de México and Instituto Nacional de Cardiología Ignacio Chávez, Mexico City 14080, Mexico; citlalli.osorio@iibiomedicas.unam.mx; 4 Departamento de Biología Molécular, Instituto Nacional de Cardiología Ignacio Chávez, Mexico City 14080, Mexico; jose.fragoso@cardiologia.org.mx; 5 Dirección de Investigación, Instituto Nacional de Cardiología Ignacio Chávez, Mexico City 14080, Mexico

**Keywords:** association study, mitochondrial DNA copy number, mitochondrial dysfunction, mitochondrial DNA, premature coronary artery disease

## Abstract

The mitochondrial DNA copy number (mtDNA-CN) is considered an indirect indicator of the number of mitochondria and of mitochondrial dysfunction; its decrease may indirectly reflect mitochondrial DNA (mtDNA) alterations. A reduction in mtDNA-CN is associated with the development of cardiovascular diseases, including coronary artery disease (CAD). The study evaluates the association of relative blood mtDNA-CN with premature CAD (pCAD) and cardiometabolic factors among Mexican individuals from the GEA (Genetics of Atherosclerotic Disease) Mexican cohort. Relative blood mtDNA-CN was quantified by real-time PCR in 835 patients with pCAD and 896 control subjects (defined as a coronary artery calcium score of zero, assessed by computed tomography). Associations were evaluated using logistic regression (odds ratio [95% confidence interval]) adjusted for potential confounders. Compared with controls, patients with pCAD exhibited significantly lower relative blood mtDNA-CN (5.6 [3.5–9.8] vs. 9.1 [5.1–13.2]. *p* < 0.001). Similar results were observed in the sex-stratified analysis. Specifically, relative blood mtDNA-CN in women (6.2 [3.8–9.9]) and men (5.5 [3.4–9.7]) with pCAD was lower than that in women (9.5 [6.3–13.7]) and men (8.2 [5.5–12.5]) from the control group (*p* < 0.001). After adjusting for age, sex, body mass index, smoking status, LDL-cholesterol, type 2 diabetes mellitus, hypertension and physical activity, higher relative blood mtDNA-CN showed a negative association with pCAD (0.903 [0.881–0.927], *p* = 4.96 × 10^−15^). This association remained significant in men (0.894 [0.866–0.922], *p* = 2.90 × 10^−12^) and women (0.917 [0.876–0.960], *p* = 2.1 × 10^−4^). Among patients with pCAD, an inverse correlation was observed between relative blood mtDNA-CN and total abdominal fat (*p* = 0.001), visceral fat (*p* = 0.015), and subcutaneous fat (*p* = 0.004). Overall, our results show that reduced relative blood mtDNA-CN is associated with pCAD, which could be indicative of mitochondrial alterations. Furthermore, the inverse correlation between relative blood mtDNA-CN and total, visceral, and subcutaneous abdominal fat suggests a link between mitochondrial dysfunction and abdominal adiposity in patients with pCAD. These findings support that decreased relative blood mtDNA-CN may serve as a marker associated with the presence of pCAD and for adipose tissue alterations in Mexican women and men.

## Simple Summary

This study examines the relationship between relative blood mitochondrial DNA copy number—a kind of “measuring stick” for cell health—and the presence of premature heart disease in Mexican individuals. Researchers compared people with premature coronary artery disease to a healthy control group. We found that those with heart problems had much lower levels of this cellular marker, suggesting their cells were not functioning as well. This result held true for both men and women. After adjusting for factors associated with cardiovascular diseases such as age, weight, cholesterol, and diabetes, we confirmed that having higher levels of this marker was associated with premature heart disease. Additionally, among patients who already had heart disease, we found an inverse relationship between this cellular marker and abdominal fat (both deep visceral fat and surface-level fat). In other words, lower cell health was associated with more belly fat. In conclusion, these results suggest that a decrease in this cellular marker could serve as an indicator of increased association with heart disease and abdominal obesity. These findings support the idea that monitoring mitochondrial health may help in the prevention and early detection of cardiovascular issues in the Mexican population.

## 1. Introduction

Cardiovascular diseases (CVDs) represent a heterogeneous group of disorders affecting the cardiac system, which comprises the heart and blood vessels [1]. They constitute a major cause of morbidity and mortality worldwide, placing an enormous burden on both patients and the healthcare system. Among these diseases, coronary artery disease (CAD) stands out, which is a consequence of the atherosclerotic process. In Mexico, CAD and its complications are the leading cause of death in men and the second leading cause in women [2]. In addition, CAD is a multifactorial disease in which both genetic and environmental factors contribute to its development. Studies in the Mexican population have identified several genetic and cardiometabolic factors associated with pCAD, supporting the relevance of investigating the biological determinants of this condition specifically in Mexican individuals [3].

Mitochondrial dysfunction and the associated oxidative stress are the cause of many chronic human diseases, including CVDs [4]. Mitochondria, while primarily acknowledged for their role in energy production, are crucial for other physiological activities, including apoptosis, redox signaling, and calcium homeostasis [5]. Recent scientific advancements have elucidated the intricate relationship between mitochondrial dysfunction and CVD. Cardiovascular complications have been associated with alterations in mitochondrial dynamics, encompassing fusion, fission, and diminished mitophagy [6,7]. The genome of the mitochondria, known as mitochondrial DNA (mtDNA), encodes 13 respiratory chain polypeptides, 22 transfer RNAs, and two ribosomal RNAs [8]. Multiple copies of mtDNA are found in the mitochondrion, and each cell can have up to 7000 mitochondria [9].

The mtDNA copy number (mtDNA-CN), defined as the ratio of mitochondrial to nuclear DNA copies, is commonly used as an indicator of mitochondrial abundance [10]. In addition, alterations in mtDNA-CN are associated with mitochondrial dysfunction and may indirectly reflect mitochondrial alterations [11]. mtDNA-CN should be regarded as a candidate biomarker that requires further investigation. [11]. Inflammation, oxidative stress, impaired oxidative phosphorylation, and mitochondrial dysfunction are the hallmarks of cardiometabolic diseases [12]. Recently, there has been interest in using the mtDNA-CN as a biomarker for chronic diseases due to its affordability and convenience of usage. Reduced mtDNA-CN has been linked to an increased presence of cardiovascular disease in some cross-sectional, case–control, prospective epidemiological studies and meta-analyses [13,14,15]. A lower mtDNA-CN has been linked in observational studies to a higher presence of diabetes [16], obesity [17], and hypertension [18].

Considering the association between mitochondrial dysfunction and the development of CVD, as well as the relationship between this dysfunction and decreased mtDNA-CN, the objective of this study was to evaluate whether there is an association between relative blood mtDNA-CN and the presence of premature CAD (pCAD) and cardiometabolic factors in Mexican individuals from the GEA (Genetics of Atherosclerotic Disease) Mexican cohort.

## 2. Materials and Methods

### 2.1. Study Population

The Genetics of Atherosclerotic Disease (GEA) study was initiated at the National Institute of Cardiology Ignacio Chávez to examine the genetic bases of pCAD and the interplay between traditional and emerging factors associated with CVD in the adult Mexican population. The study comprised 1240 patients diagnosed with CAD and 1500 individuals without a family history or clinical manifestations of pCAD, aged 30 to 75 years. All participants are unrelated Mexican mestizos, each possessing a lineage of no fewer than three generations born in Mexico. CAD was defined by a history of myocardial infarction, angioplasty, revascularization surgery, or coronary stenosis greater than 50% as assessed by angiography. pCAD was considered when diagnosis was made before the age of 55 in males and 65 in females. People who had an acute cardiovascular event in the last three months were not allowed to take part in the study. Participants in the control group, who had no family history or clinical signs of pCAD, were selected from individuals attending the Institute’s blood bank or recruited via written invitation. People who had a history or proof of liver, kidney, cancer, or thyroid diseases were not allowed to take part in the study.

All participants answered standardized questionnaires to collect demographic data, family and personal history of factors associated with CVD, dietary habits, physical activity, alcohol consumption, and medication usage, adhering to established methodologies [19]. To assess the effects of population stratification, in all participants we analyzed 265 ancestry-informative markers distinguishing Amerindian, European, and African ancestry, as detailed by HapMap [20]. The proportions of Caucasian, Amerindian, and African ancestry were evaluated using the ADMIXTURE program in conjunction with previously recorded data for these populations. The overall ancestry of both study groups was similar: 54.0% Amerindian, 35.8% Caucasian, and 10.1% African in the control group, and 55.8% Amerindian, 34.3% Caucasian, and 9.8% African in the patients with pCAD (*p* > 0.05 for all comparisons).

**Figure 1:**
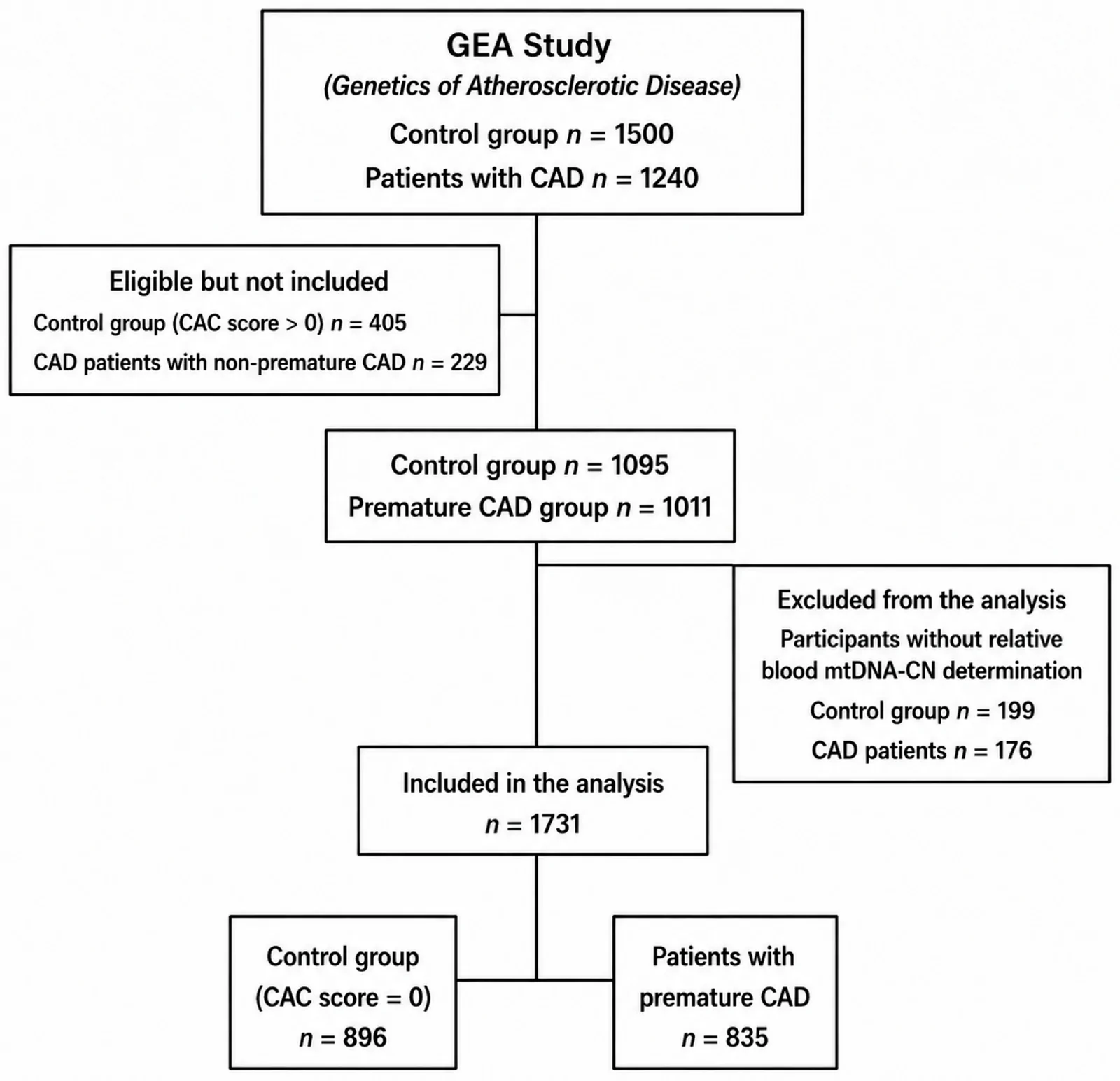
Flow chart representing the distribution of the participants in the study.

In this study, 896 participants were selected from a control group of 1500 individuals, all demonstrating no indications of subclinical atherosclerosis (coronary artery calcium score = 0). Furthermore, 835 patients with pCAD were incorporated, for whom determination of mtDNA-CN was available (Figure 1).

### 2.2. Anthropometric Measurements

Trained personnel measured weight in kilograms (kg) and height in centimeters (cm), using a calibrated scale and a wall-mounted stadiometer. Body mass index (BMI) was calculated using the formula weight (kg)/height (m^2^). Waist circumference was measured with a fiberglass measuring tape at the midpoint between the lower edge of the last rib and the iliac crest.

### 2.3. Laboratory Analysis

After a 10–12-h fast, venous blood samples were collected in a seated position. Plasma concentrations of glucose, total cholesterol, triglycerides, and high-density lipoprotein cholesterol (HDL-C) were determined by enzymatic–colorimetric methods (Roche/Hitachi, Mannheim, Germany) on a Hitachi 902 automated analyzer (Hitachi Ltd., Tokyo, Japan). Low-density lipoprotein cholesterol (LDL-C) concentrations were estimated using the Friedewald formula [21]. The reproducibility and accuracy of lipid determinations in our laboratory were evaluated by the Centers for Disease Control and Prevention in Atlanta (CDC, Atlanta, GA, USA). The intra- and inter-assay coefficients of variation were less than 3%.

### 2.4. Definition of Hypertension and Type 2 Diabetes Mellitus

After a 10 min rest, systolic and diastolic blood pressures were measured. The average of the second and third measurements was recorded for analysis. We considered hypertension as self-reported treatment with antihypertensive medications or a systolic blood pressure ≥ 140 mm Hg and/or diastolic blood pressure ≥ 90 mm Hg. Type 2 diabetes mellitus was defined using the American Diabetes Association criteria [21]. It was also considered when participants reported glucose-lowering treatment, or a physician diagnosis of diabetes.

### 2.5. Computed Axial Tomography Study

Multidetector computed tomography (MDCT) is a useful, non-invasive, and widely validated method for quantifying coronary artery calcium (CAC), total abdominal fat (TAF), subcutaneous abdominal fat (SAF), and visceral abdominal fat (VAF) in a single session. In this study, chest and abdominal images were acquired using a 64-slice CT scanner (Somaton Sensation, Forchheim, Germany). The tomographic images were interpreted by experienced radiologists to determine and quantify: (1) VAF, SAF, and TAF [22]; and (2) the CAC score using the Agatston method [23]. To define the cutoff points indicating increased TAF, VAF, and SAF deposition, a subsample of 131 men and 185 women from the GEA control group database was selected; these individuals were non-obese and had normal lipid, glucose, and blood pressure levels. An increase in the studied adipose tissue deposits was considered when values were at or above the 75th percentile for men and women. An increase in TAF was considered when values were ≥462.50 cm^2^ in women and ≥373.25 cm^2^ in men; for VAF, the values were ≥122.00 cm^2^ in women and ≥151.50 cm^2^ in men; and for SAF, they were ≥335.5 cm^2^ in women and ≥221.75 cm^2^ in men. The presence of subclinical coronary atherosclerosis (AS) was defined as a CAC score greater than 0 [24,25].

### 2.6. Determination of mtDNA-CN

We adapted a multiplex quantitative real-time polymerase chain reaction method with minor modifications [26,27]. Genomic DNA was extracted from whole peripheral blood using the QIAamp DNA Blood Mini Kit (QIAGEN, Hilden, Germany), without prior separation of leukocytes. To measure relative mtDNA content, we used 8 ng of DNA and the probe (6FAM-5′ TAAGATGGCAGAGCCCGGTAATCGCAT-3′-MGB). The primer sequences used for mtDNA amplification were mtF3212 (5′ CACCCAAGAACAGGGTTTGT 3′) and mtR3319 (5′ TGGCCATGGGTRTGTTGTTAA 3′).

The amount of mtDNA was normalized by simultaneously measuring a single-copy nuclear gene for ribonuclease P (TaqMan RNase P Control Reagents Kit, ThermoFisher Scientific, Waltham, MA, USA). Real-time polymerase chain reaction assays were performed using the QuantStudio 12K Flex Real-Time PCR System (ThermoFisher Scientific, Waltham, MA, USA). The qRT-PCR cycling conditions were as follows: 50 °C for 2 min, 95 °C for 10 min, followed by 40 cycles of 95 °C for 15 s and 60 °C for 1 min.

A laboratory reference DNA sample prepared as a pool of 20 study samples was used for normalization. Each plate contained a five-point calibration curve (10, 3.33, 1.11, 0.37, and 0.12 ng/μL), which was used to estimate mtDNA and nDNA quantities. For each test sample, relative mtDNA copy number (mtDNA-CN) was calculated as the mtDNA/nDNA ratio standardized to the mtDNA/nDNA ratio of the reference DNA pool. Thus, a relative mtDNA-CN value of 1 indicates that the mtDNA/nDNA ratio of the test sample is equal to that of the reference DNA pool. Accordingly, the mtDNA-CN values reported in this study represent relative mitochondrial DNA content rather than absolute numbers of mitochondrial genome copies per cell. All samples and standards were analyzed in triplicate. The investigator performing the qPCR assays was aware of whether samples belonged to the pCAD or control group but had no access to any additional participant-level clinical information during the laboratory analyses. Samples were analyzed in the order in which they were provided to the laboratory and were not randomized by case–control status before qPCR analysis.

### 2.7. Statistical Analysis

Categorical variables are expressed as proportions, and continuous variables as mean ± standard deviation or median (interquartile range). Group comparisons were performed using the chi-square test for categorical variables, Student’s t-test for parametric continuous variables, and the Mann–Whitney U test for nonparametric continuous variables. The general characteristics are shown for the study population stratified by the presence or absence of pCAD.

The analysis of relative blood mtDNA-CN in the study groups, stratified by sex, was performed using Mann–Whitney U tests. A sex-by-relative blood mtDNA-CN interaction test was made.

Multivariate logistic regression analysis [odds ratio (OR) and 95% confidence interval (CI)] was used to determine the independence of the association between relative blood mtDNA-CN and the presence of pCAD, adjusted for age, sex, body mass index, smoking status, LDL-cholesterol, type 2 diabetes mellitus, hypertension and physical activity. All analyses were performed using the SPSS v15.0 statistical package (SPSS, Chicago, IL, USA). Differences were considered statistically significant at *p* < 0.05.

We performed a statistical power analysis using an independent external effect estimate from [28]. In their combined cohort, the association between relative blood mtDNA-CN and coronary artery disease was reported as an OR of 1.16 (95% CI: 1.08–1.25) per 1-SD decrease in relative blood mtDNA-CN. Using this effect estimate, a two-sided α of 0.05, and the case-to-control ratio observed in the GEA study (835 pCAD cases and 896 controls; N = 1731), the estimated statistical power was 87.0%.

## 3. Results

### 3.1. Clinical, Biochemical, and Adiposity Characteristics

The general characteristics of the study population are shown in Table 1. In patients with pCAD, age, percentage of males, BMI, waist circumference, triglyceride concentration, and VAT volume were significantly higher than those in control subjects (*p* < 0.01 for all comparisons). Due to the effect of statin treatment, total cholesterol and LDL-C concentrations in coronary patients were significantly lower than those in control subjects (*p* < 0.001 for all variables). HDL-C concentrations were significantly lower in patients with pCAD than those in control subjects (*p* < 0.001). Hypertension and type 2 diabetes mellitus were significantly more prevalent in pCAD patients compared to controls (*p* < 0.001).

### 3.2. Relative Blood mtDNA-CN in the Study Groups

**Figure 2:**
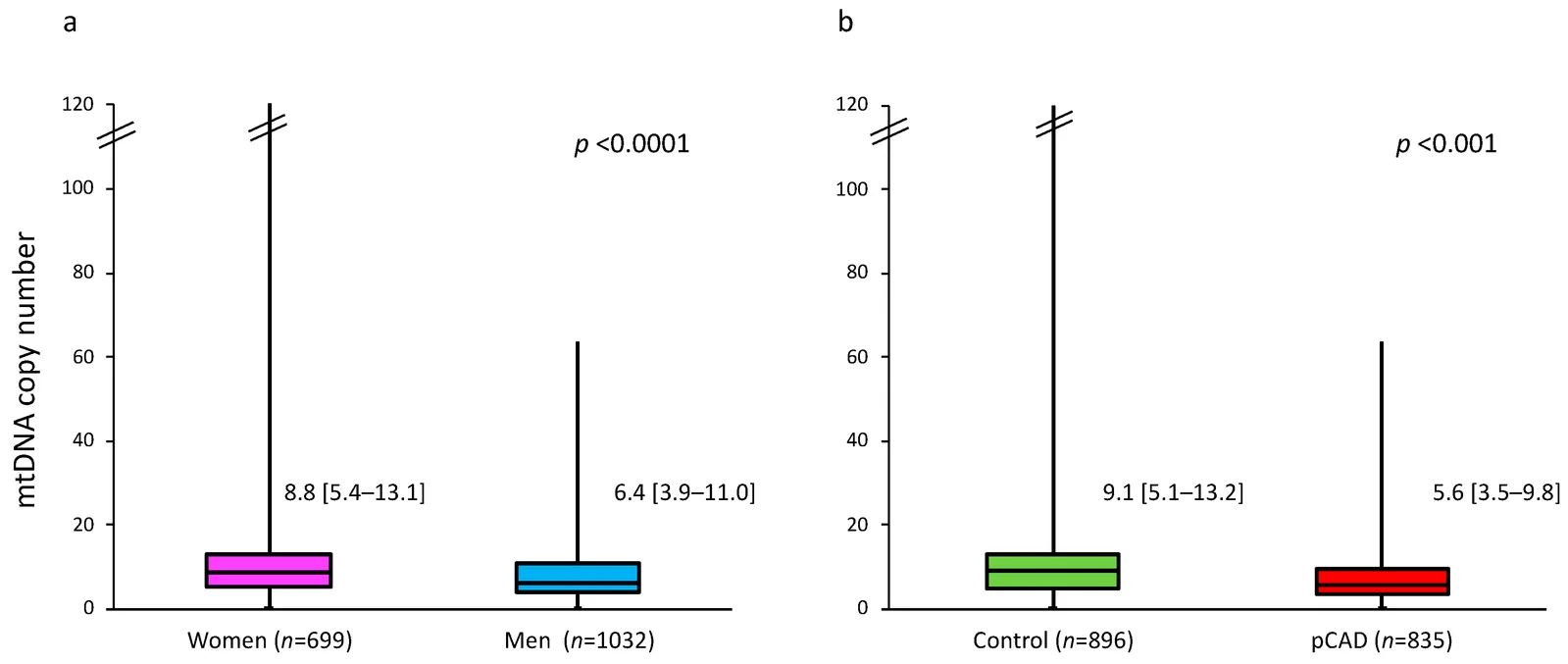
Relative blood mtDNA-CN in the study population stratified by sex (**a**) and studied groups (**b**). Data is expressed as median [interquartile range]. Comparisons between groups were made with Mann–Whitney U test.

The median of relative blood mtDNA-CN in the entire study population (*n* = 1731) was 7.3 [4.5–11.8]. When stratifying the study population by sex, men (*n* = 1032, 6.4 [3.9–11.0]) had lower relative blood mtDNA-CN compared with women (*n* = 699, 8.8 [5.4–13.1]) (*p* < 0.0001). Group analysis showed that, compared with individuals in the control group, patients with pCAD have lower relative blood mtDNA-CN (9.1 [5.1–13.2] vs. 5.6 [3.5–9.8], *p* < 0.001) (Figure 2a,b).

**Figure 3:**
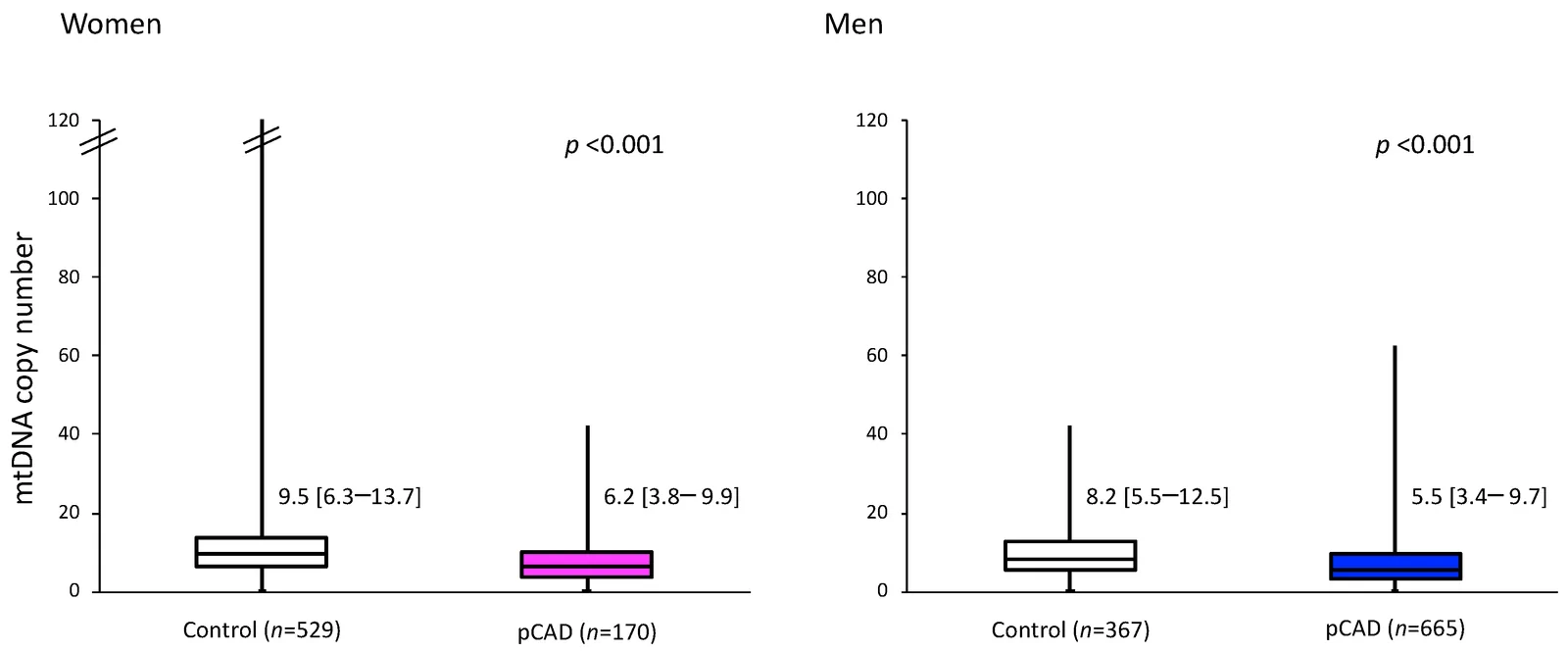
Relative blood mtDNA-CN in women and men stratified by studied groups. Data is expressed as median [interquartile range]. Comparisons between groups were made with Mann–Whitney U test.

Similar results were observed in the sex-stratified analysis. The relative blood mtDNA-CNs in women (9.5 [6.3–13.7]) and men (8.2 [5.5–12.5]) in the control group were higher than those in women (6.2 [3.8–9.9]) and men (5.5 [3.4–9.7]) with pCAD (*p* < 0.001) (Figure 3).

**Figure 4:**
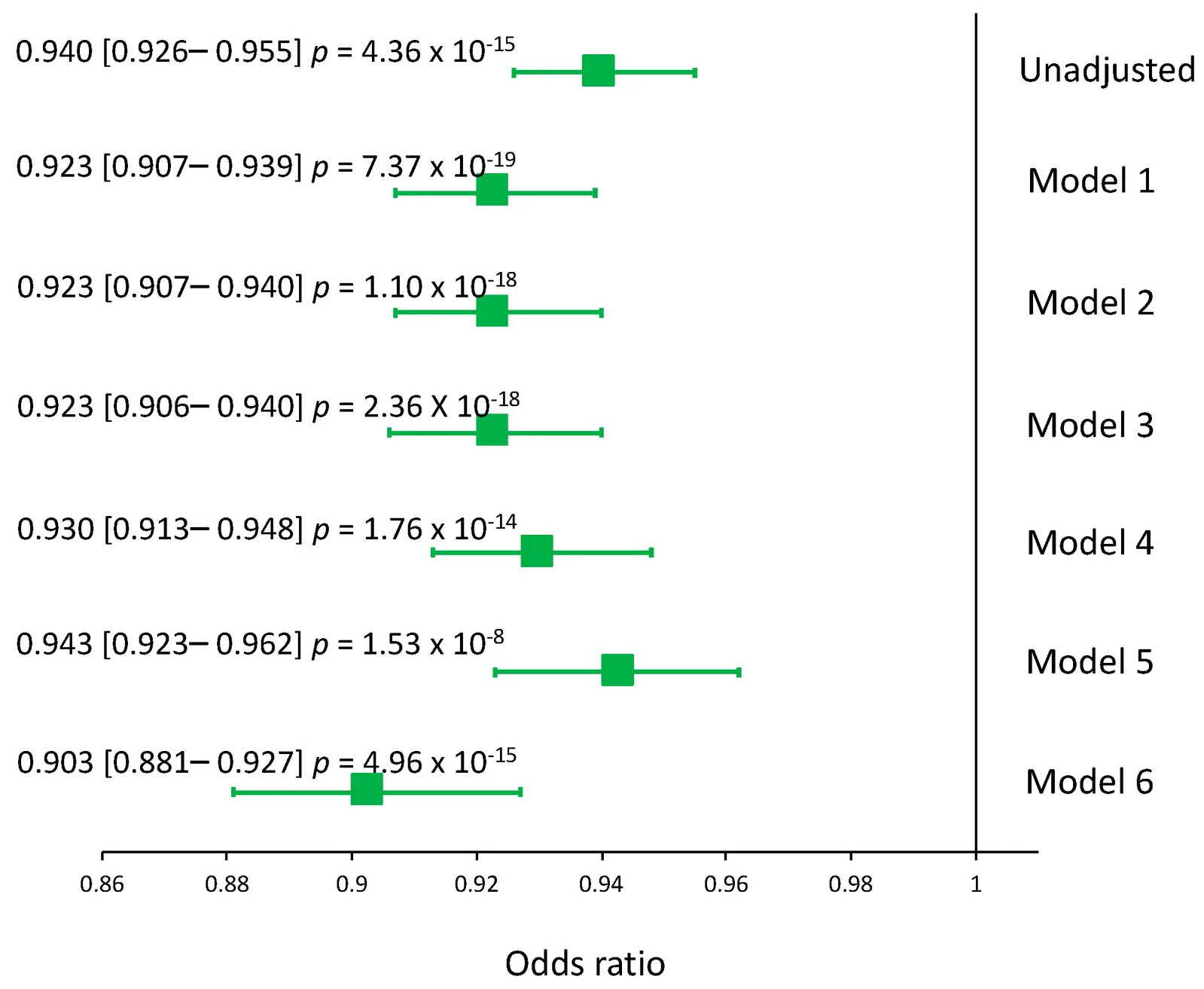
Association between pCAD and relative blood mtDNA-CN in the study population. Model 1: Adjusted for age and sex; Model 2: Adjusted for model 1 + body mass index; Model 3: Adjusted for model 2 + current smoking; Model 4: Adjusted for model 3 + LDL-cholesterol; Model 5: Adjusted for model 4 + type 2 diabetes mellitus and hypertension; Model 6: Adjusted for model 5 + physical activity.

Logistic regression analysis, adjusted for age, sex, BMI, smoking status, LDL-C, type 2 diabetes mellitus, hypertension and physical activity (model 6), demonstrated that higher relative blood mtDNA-CN was significantly associated with reduced pCAD (0.903 [0.881–0.927], *p* = 4.96 × 10^−15^) (Figure 4).

**Figure 5:**
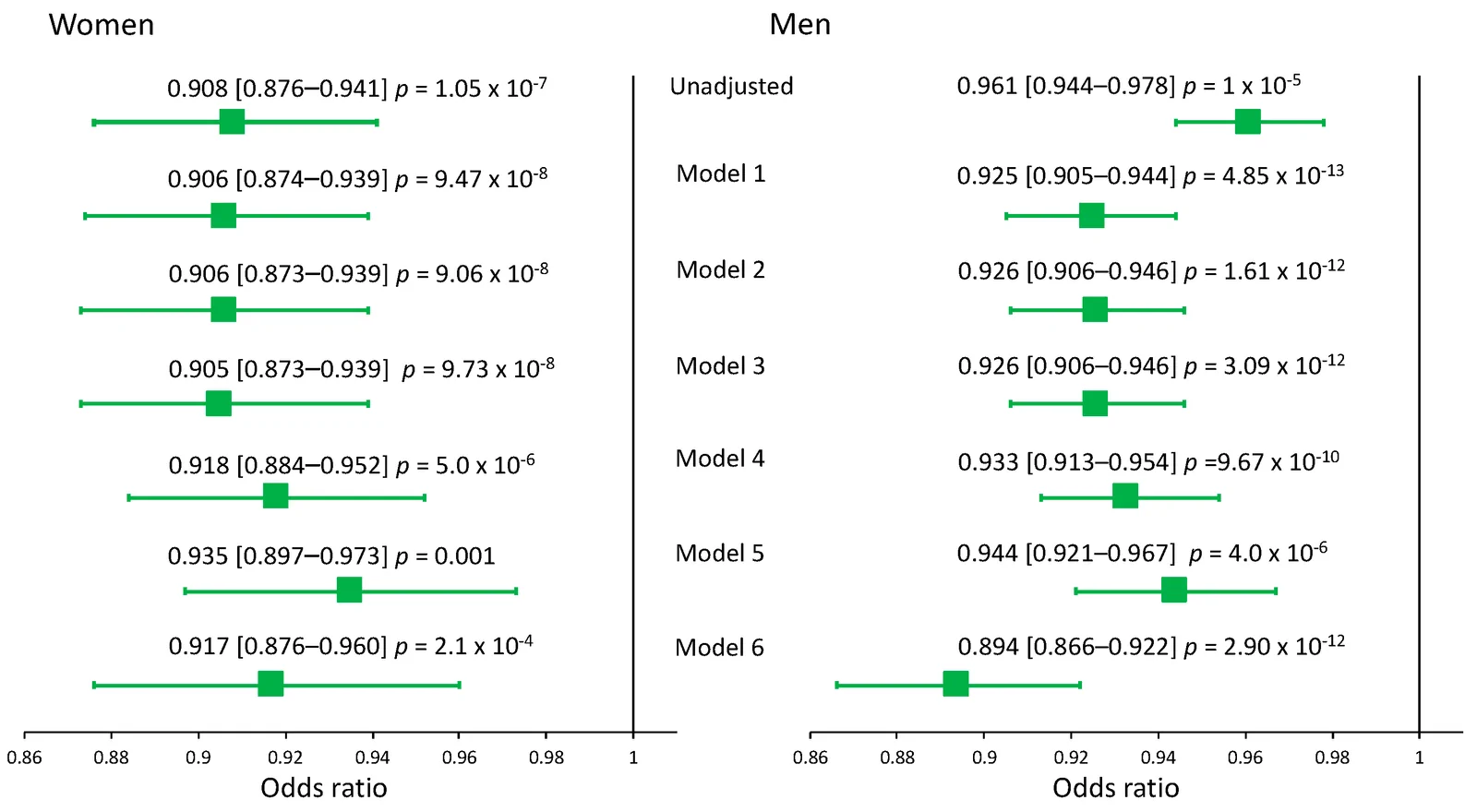
Association between pCAD and relative blood mtDNA-CN in the study population stratified by sex. Model 1: Adjusted for age; Model 2: Adjusted for model 1 + body mass index; Model 3: Adjusted for model 2 + current smoking; Model 4: Adjusted for model 3 + LDL-cholesterol; Model 5: Adjusted for model 4 + type 2 diabetes mellitus and hypertension; Model 6: Adjusted for model 5 + physical activity.

The association remained significant when the analysis was performed in women (0.917 [0.876–0.960], *p* = 2.1 × 10^−4^) and men (0.894 [0.866–0.922], *p* = 2.9 × 10^−12^) (Figure 5). However, the interaction term was not statistically significant (*p* = 0.597), indicating that the association between relative blood mtDNA-CN and pCAD does not differ significantly between men and women.

**Figure 6:**
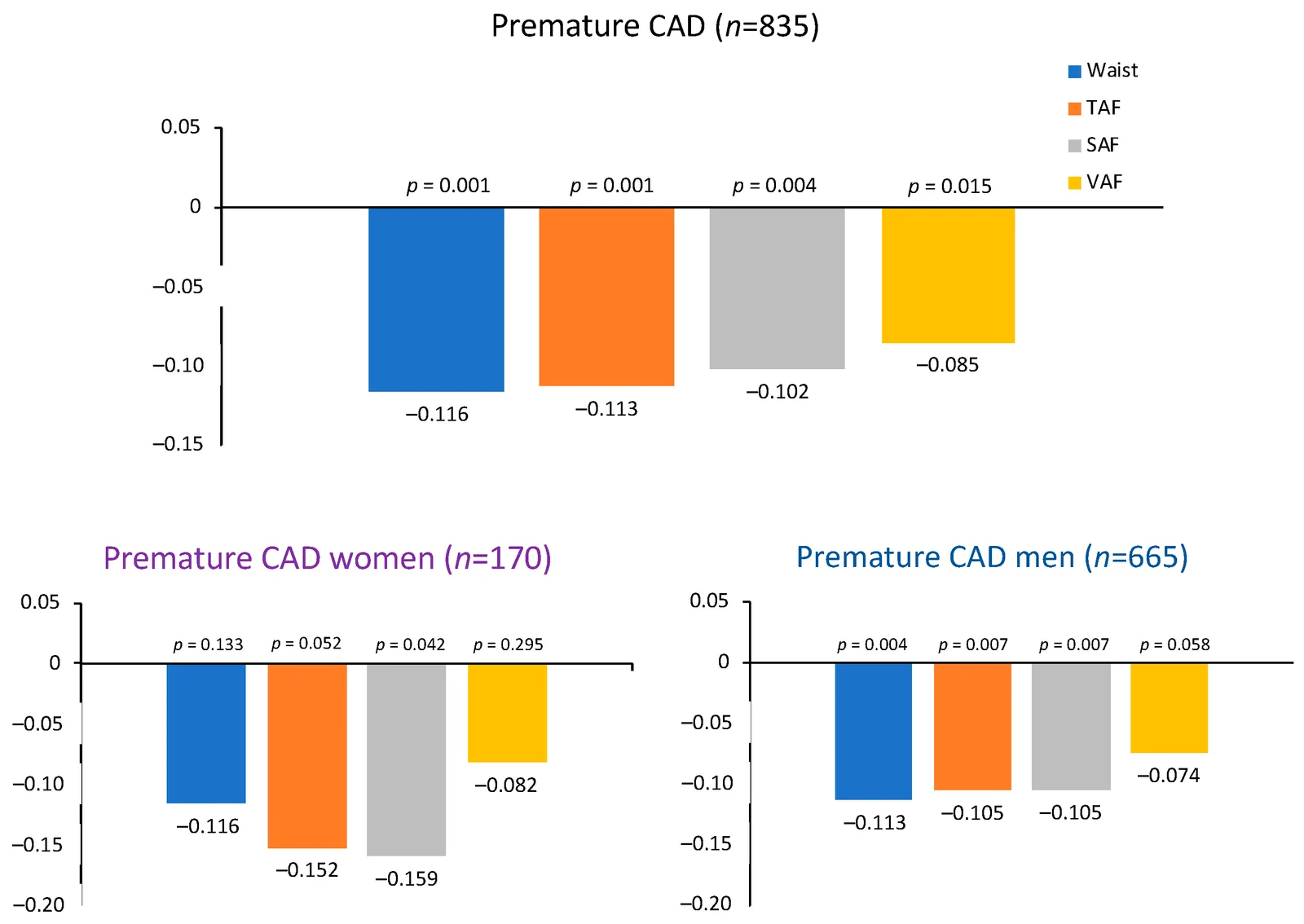
Spearman correlation between relative blood mtDNA-CN and adiposity measurements in the premature coronary patients. TAF = total abdominal fat, SAF = subcutaneous abdominal fat, VAF = visceral abdominal fat. The *p* values were not adjusted for multiple comparisons because these were simple bivariate correlations testing specific predefined hypotheses.

In patients with pCAD, an inverse correlation was observed between relative blood mtDNA-CN and waist circumference (*p* = 0.001), TAF (*p* = 0.001), SAF (*p* = 0.004), and VAF (*p* = 0.015) (Figure 6). When the analysis was performed in men and women with pCAD, an inverse and significant correlation was observed in men between relative blood mtDNA-CN and waist circumference (*p* = 0.004), TAF (*p* = 0.007), and SAF (*p* = 0.007) (Figure 6). In women, SAF showed a significant inverse correlation with relative blood mtDNA-CN (*p* = 0.042) (Figure 6).

## 4. Discussion

The results of this study offer pertinent evidence regarding the potential role of relative blood mtDNA-CN as a biomarker associated with the presence of pCAD. Initially, it was noted that patients with pCAD exhibited diminished relative blood mtDNA-CN relative to controls, showing a 38% decrease in the median. This reduction remained consistent when analyzed by sex, with a 35% decrease in women and a 36% decrease in men, indicating that the finding is reliable and not influenced by sex. These findings align with international research that associates low relative blood mtDNA-CN with cardiovascular diseases [13,15]. The decrease in relative blood mtDNA-CN may indicate mitochondrial dysfunction, a condition previously documented in diverse cardiovascular disease contexts [29]. This discovery corroborates the hypothesis that compromised mitochondrial function is a pivotal mechanism in the pathophysiology of atherosclerosis and its associated clinical complications. After controlling for possible confounding variables, individuals with elevated relative blood mtDNA-CN exhibited a diminished association with pCAD. In quantitative terms, for every unit increase in relative blood mtDNA-CN, the presence of pCAD diminished by approximately 9.7% in the total group (10.6% in men and 8.3% in women), with no evidence of a differential association by sex (*p* for interaction = 0.597). Previous studies conducted in populations from different countries have similarly reported an inverse association between mtDNA-CN and cardiovascular disease. In a large prospective analysis including participants from the Atherosclerosis Risk in Communities (ARIC), Cardiovascular Health Study (CHS), and Multi-Ethnic Study of Atherosclerosis (MESA) cohorts in the United States, lower mtDNA-CN was associated with a higher presence of incident coronary heart disease and cardiovascular disease, with consistent associations across sex and race/ethnicity groups [13]. Similarly, a two-stage case–control study of 3064 Chinese individuals found that each 1-SD decrease in mtDNA-CN was associated with a higher presence of CAD (14% higher odds), and lower mtDNA-CN was also associated with greater disease severity [28]. More recently, a systematic review and meta-analysis of observational studies reported that individuals with the lowest mtDNA-CN had a higher presence of cardiovascular disease and coronary heart disease compared with those with the highest mtDNA-CN (RR 2.09 and 1.70, respectively) [30]. Together with our findings in Mexican individuals with pCAD, these data suggest that the inverse association between mtDNA-CN and cardiovascular disease may be observed across diverse populations, although differences in study design, population characteristics, and methods used to quantify mtDNA-CN should be considered. However, mitochondrial DNA copy number is a non-specific biomarker, and its fluctuations are linked to a wide range of pathologies. Low mtDNA-CN is associated with type 2 diabetes and metabolic syndrome [31]. Cross-sectional analyses from the UK Biobank (over 400,000 participants) further demonstrated that lower mtDNA-CN is independently associated with higher odds of obesity, hypertension, diabetes, and hyperlipidemia [32]. These findings underscore the potential of mtDNA-CN as a valuable biomarker for stratifying cardiometabolic associations.

Also, statin therapy has been shown to exert significant but complex effects on mitochondrial DNA (mtDNA) copy number, with studies reporting both increases and decreases depending on the specific drug, cell type, and patient age [13]. Atorvastatin and simvastatin elevated mtDNA copy numbers in rat astrocytes, while rosuvastatin increased mtDNA content in cortical neurons following ischemic injury [33]. Mendelian randomization analyses in humans have linked statin use to a decline in mtDNA copy number that may contribute to memory loss [34]. These effects are mechanistically attributed to statin-induced inhibition of the mevalonate pathway, which reduces coenzyme Q10 synthesis and increases mitochondrial oxidative stress. Additionally, lovastatin has been shown to induce mitochondrial ROS generation and direct alterations in mtDNA [35]. The bidirectional nature of these responses highlights the need for further research to clarify the clinical implications, particularly regarding long-term statin use and mitochondrial health. Emerging evidence also suggests that mitochondrial genetic background and mtDNA-CN could serve as potential indicators for predicting individual association with statin-related side effects and cardiovascular outcomes [36].

The association between diminished relative blood mtDNA-CN and heightened presence of pCAD underscores the notion that mitochondria are crucial for sustaining vascular cellular homeostasis. Oxidative stress, mutations in mtDNA, and cellular repair failures are the main causes of mitochondrial alterations [37]. These alterations change how mitochondria normally work, which causes mitochondrial dysfunction. These changes affect the ATP and cause too many reactive oxygen species to be made, which leads to inflammation [38].

**Figure 7:**
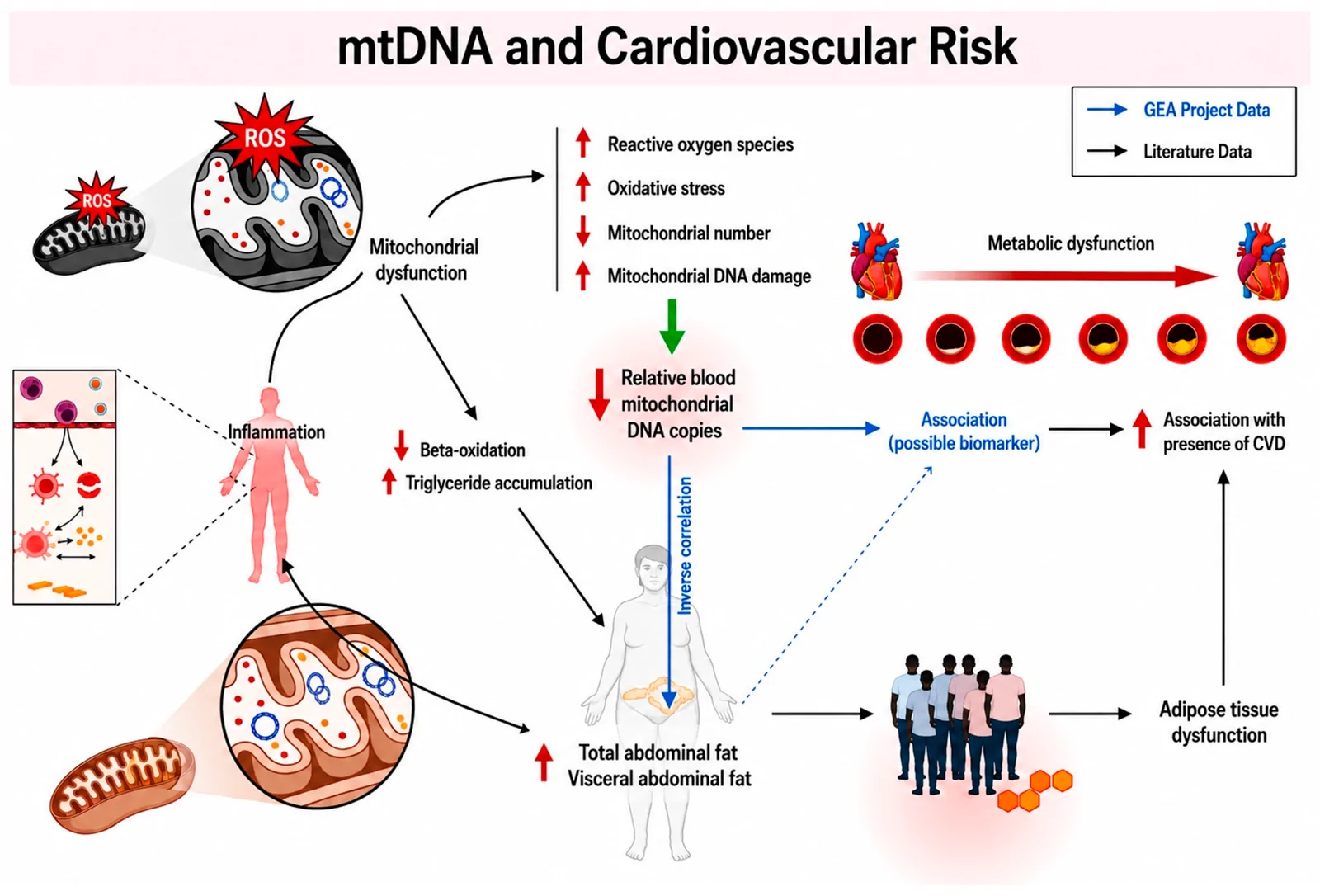
Conceptual hypothesis. An inverse correlation between relative blood mtDNA-CN and both total abdominal adiposity and, more specifically, visceral adipose tissue (VAT), was observed. VAT is known to secrete proinflammatory cytokines while simultaneously reducing the production of adiponectin, an adipokine with protective effects on mitochondrial function. These inflammatory signals interfere with mitochondrial biogenesis, thereby promoting mitochondrial dysfunction. Such dysfunction leads to increased generation of reactive oxygen species (ROS) and elevated oxidative stress, which further contribute to a reduction in mitochondrial mass, greater mitochondrial alterations, and a subsequent decrease in relative blood mtDNA-CN. These interconnected processes may contribute to visceral fat accumulation, although this proposed relationship cannot be established from the present study. Our findings demonstrate that reduced relative blood mtDNA-CN is significantly associated with pCAD and correlates inversely with total and visceral abdominal fat depots. Consequently, we propose that relative blood mtDNA-CN may warrant further evaluation as a useful indicator associated with the presence of cardiovascular disease.

In the cardiovascular domain, mitochondrial dysfunction compromises the viability of endothelial cells, cardiomyocytes, and vascular smooth muscle cells. This leads to oxidative stress, cell death, and aging [39]. These processes facilitate atherosclerosis, hypertension, and cardiac remodeling, thereby advancing the development and progression of cardiovascular disease. mtDNA-CN is regarded as an indirect measure of mitochondrial quantity and dysfunction; its reduction may indirectly signify mitochondrial alterations [40]. A decrease in mtDNA-CN has been linked to the development of cardiovascular diseases, including coronary artery disease [14,15,28,41]. A decrease in mtDNA-CN leads to a compromised ability to maintain mitochondrial biogenesis and sufficient energy production, promoting a pro-oxidative and inflammatory condition that leads to endothelial injury, smooth muscle cell impairment, and progression of atherosclerotic plaque [42,43]. The findings also indicate an inverse relationship between relative blood mtDNA-CN and TAF, SAF, and VAF. This relationship is significant because visceral adipose tissue correlates with increased systemic inflammation [44], insulin resistance [45], and oxidative stress [46], all of which can directly impact mitochondrial integrity and quantity. Research involving Mexican–American populations has established a correlation between adiposity and a reduced mtDNA-CN [47]. In this manner, the decrease in mtDNA-CN may function as an initial indicator of metabolic and cardiovascular impairment, as well as a pathway through which visceral obesity elevates the presence of pCAD. Mitochondrial dysfunction and excessive visceral fat are interrelated processes that influence one another. Mitochondria are responsible for beta-oxidation; thus, mitochondrial dysfunction diminishes their ability to oxidize lipids, resulting in the accumulation of fatty acids as triglycerides in visceral adipose tissue. Conversely, the accumulation of VAF induces inflammation that compromises mitochondrial integrity, resulting in mitochondrial dysfunction. This dysfunction leads to an elevation in free radicals and oxidative stress, resulting in a reduction in the number of mitochondria and alterations to mtDNA; these alterations are directly correlated with a decrease in mtDNA-CN. Our findings indicate that the reduction in relative blood mtDNA-CN may represent a pathophysiological connection among central obesity, mitochondrial dysfunction, and the development of pCAD. Additionally, they represent the potential to regard relative blood mtDNA-CN as an attainable biomarker for cardiovascular disease stratification in populations characterized by a significant prevalence of obesity and pCAD, exemplified by the Mexican population (Figure 7). However, these findings are based on weak cross-sectional correlations and have therefore been interpreted cautiously, as they do not support conclusions about adipose tissue dysfunction or establish a mechanistic link between abdominal adiposity, mitochondrial alterations, and pCAD. The proposed pathway remains speculative and requires further mechanistic and longitudinal studies to be validated.

Our study has some strengths. First, it has a large group of Mexican cases and controls that are well-defined biochemically, clinically, and by imaging. This lets us adjust the analyses for many possible confounders. The adjustments mentioned above enable us to isolate the effect of relative blood mtDNA-CN from other known factors. Secondly, the control group consists solely of individuals devoid of tomographic evidence of subclinical atherosclerosis (CAC score = 0), rendering this group free from the studied pathology and thus the optimal control. Lastly, while relative blood mtDNA-CN is not directly genetically determined, DNA polymerase and the mitochondrial transcription factor control how many copies are made and kept. Changes in the genes that code for these proteins can raise or lower relative blood mtDNA-CN [48,49]. Adjustment for ancestry markers may help reduce potential confounding due to population stratification. Nevertheless, it is crucial to recognize specific limitations. Being a case–control study, it is impossible to determine a causal relationship between the decrease in relative blood mtDNA-CN and pCAD. The results are consistent and have been adjusted for factors associated with CVD; however, residual confounding by unassessed variables, including diet, or metabolic comorbidities, cannot be excluded. The use of blood bank volunteers as controls is a potential limitation. However, the control group was established through a rigorous community-based sampling strategy that included blood donors, their parents, social service users, and volunteers recruited through open invitations. Retaining donors in the analysis enabled us to capture a broad spectrum of cardiovascular health, from optimal physiological status to the community baseline. This multi-channel approach ensured that controls shared the same geographical area as cases, while the inclusion of parents and older volunteers corrected the age disparity by providing middle-aged and elderly participants. The resulting heterogeneity effectively diluted the “healthy donor effect,” offering a realistic population reflection. It is important to note that DNA was extracted from whole blood rather than from isolated leukocytes. Consequently, the mtDNA-CN values obtained may be influenced by inter-individual variations in blood cell composition (e.g., differential leukocyte counts and platelet counts), which were not available for our cohort. Similarly, environmental, genetic, and epigenetic factors unique to the Mexican cohort may influence both relative blood mtDNA-CN and the presence of pCAD, highlighting the necessity for longitudinal and functional studies to validate these associations.

## 5. Conclusions

In conclusion, the reduction in the relative blood mtDNA-CN is significantly associated with the occurrence of pCAD and abdominal adiposity. These findings add to the evidence supporting an association between relative mtDNA-CN in pCAD and proposing novel avenues for identifying possible indicators and potential therapeutic targets in this condition.

## Figures and Tables

**Table 1:** Characteristics of the studied groups.

	Controls	Premature CAD	*p*
N	896	835	
Age (years)	51 ± 9	52 ± 9	0.006
Sex (% of men)	41.0	79.6	<0.001
Body Mass Index (kg/m^2^)	27.9 [25.4–31.0]	28.4 [26.1–31.2]	0.004
Waist circumference (cm)	94 ± 11	98 ± 10	<0.001
Total cholesterol (mg/dL)	190 [168–211]	162 [133–194]	<0.001
HDL-cholesterol (mg/dL)	45 [36–55]	37 [31–44]	<0.001
LDL-cholesterol (mg/dL)	116 [97–136]	92 [70–117]	<0.001
Triglycerides (mg/dL)	142 [106–202]	165 [118–223]	<0.001
Glucose (mg/dL)	90 [84–98]	94 [86–115]	<0.001
Total physical activity	7.9 [7.0–8.8]	7.5 [6.8–8.5]	<0.001
Abdominal fat			
Total (cm^2^)	434 [350–536]	428 [340–522]	0.177
Visceral (cm^2^)	140 [105–182]	166 [130–209]	<0.001
Subcutaneous (cm^2^)	291 [219–370]	251 [194–319]	<0.001
Increased abdominal fat			
Total (%)	54.2	60.8	0.006
Visceral (%)	56.0	65.3	<0.001
Subcutaneous (%)	49.8	55.2	0.028
Type 2 diabetes mellitus (%)	10.5	33.2	<0.001
Hypertension (%)	19.3	67.3	<0.001
Current smoking (%)	23.1	12.5	<0.001
Lipid lowering treatment	7.7	94.1	<0.001
Hypertensive treatment	12.4	97.8	<0.001

Data are shown as mean ± standard deviation, median [interquartile range], or percentages. Comparisons between groups were performed using Student’s t, Mann–Whitney U, or Chi-square tests, depending on the type of variable.

## Data Availability

Data will be made available on request.
